# Prize Sessions

**Published:** 2008-10

**612/ AP01 G Achari Prize**

**Studies on the prescribing pattern of antibiotics and multi-vitamins in university health centre**

Aggarwal A, Kumar A

**Panjab University, Chandigarh, India.**

**Objective:**

Present study was aimed to establish the prescribing pattern of antibiotics as well as multivitamin in the University Health Centre in order to promote rational use of drugs.

**Patients and Methods:**

The study was conducted at University Health Centre, by using WHO based Prescription auditing Performa that includes- Patients related information (name, age, sex, disease diagnosed), drug related information (name, brand/ generic, individual/ combination, dose, frequency, availability of medicine, dosage form) and prescribing instruction for pharmacist (oral/ written, dispensing time; patient knowledge- use) and nature of medication by interviewing OPD patients (n=200 patients).

**Results:**

In the present study, 200 prescriptions were monitored that contained the age, gender and name of the patients and 530 drugs in total. Most medicines were dispensed from University Health Centre. However, diseases diagnosed were not mentioned in majority of prescriptions-184 (92%). Antibiotics and multivitamins were prescribed 162 (30.56 %) and 139 (26.25%) respectively. Major antibiotics were amoxicillin 52(30.10%), doxycycline 34 (21.10%) and ciprofloxacin 24 (14.8%). Similarly, major multivitamin prescribed were becozyme 66 (47.5%), ferritop 16 (11.5%) and neurobion inj 15(10.7%). Individual 280 (53%) and drug in combinations 250 (47%) were equally prescribed. Generic drugs were more prescribed 344(65%) in comparison to branded 118(35%). Average consulting time, dispensing time, no. of drugs per prescription and no. of patients in OPD per day seen by the doctor were 4.5 minutes, 1.5 minutes, 3 and 256, respectively.

**Conclusion:**

There are number of lacunae in the present prescribing practice which can be improved by further interventional studies.

**613/ AP02 G Achari Prize**

**Effects of add-on therapy with insulin sensitizers in patients of type 2 diabetes mellitus on dual drug therapy.**

Arora P^1^, Singh J^2^

**^1^M.M Institute of Medical Sciences & Research, Mullana (Ambala), ^2^Government Medical College, Amritsar, India.**

Besides the well established effects of thiazolidinediones on glycemic control and blood lipid levels, they also hold promise to improve beta cell function and also decrease cardiovascular events. Aim was to see the effectiveness and safety of add-on therapy with rosiglitazone and pioglitazone in combination with glibenclamide and metformin in uncontrolled type 2 diabetic patients. 60 patients uncontrolled on glibenclamide (5-15 mg)/metformin (500mg) for 6 months were recruited in this 24 weeks long randomized placebo controlled study. Divided into three groups of 20 patients each, addon therapy was given with rosiglitazone (4 mg), Pioglitazone (15 mg) and a placebo respectively. Levels of HbA1c, total cholesterol, triglycerides, HDL, LDL, fasting insulin, C-peptide and CRP levels were recorded and compared. Addition of rosiglitazone & pioglitazone showed a significant decrease in HbA1c by 1% each. Treatment with rosiglitazone and pioglitazone showed an increase in mean levels of HDL by 6.0 mg/dl & 7.2 mg/dl, and a decrease in the levels of triglycerides by 43.5 mg/dl & 42.3 mg/dl, fasting plasma insulin by 9.0 mg/dl & 7.2 mg/dl, C-peptide by 0.5 mg/dl each and CRP levels by 0.13 mg/dl & 0.14 mg/dl respectively from the baseline. Rosiglitazone was associated with increased levels of total cholesterol by 31.4 mg/dl, LDL by 34.1 mg/dl & pioglitazone with a decrease in the mean levels of total cholesterol by 53.2 mg/dl, LDL by 52 mg/dl. The effects were statistically significant. No serious adverse effects occurred requiring discontinuation of therapy. Addition of insulin sensitizers to dual drug therapy produces better glycemic control & also results in improved levels of insulin & cardiovascular risk markers.

**614/AP 03 G Achari Prize**

**Evaluation of anti-inflammatory potential of rosiglitazone, a peroxisome proliferator activated receptor gamma agonist in three doses in chronic experimental model of inflammation **

Borkar SS^1^, Manjrekar NA^2^

**^1^Seth G.S. Medical College & KEM Hospital, Mumbai, India, ^2^TNMC & Nair Hospital, Mumbai, India.**

**Introduction:**

Rosiglitazone is a specific high-affinity ligand for PPAR gamma, currently used in diabetes mellitus. The acute anti-inflammatory effects of PPAR gamma have been shown in few studies. Anti-inflammatory effects of rosiglitazone may have implication in prevention of various macro & micro vascular complications of diabetic subjects Hence we studied the effects of rosiglitazone in animal model of chronic inflammation.

**Methods:**

Anti-inflammatory effects of Rosiglitazone in the dose of 1,3,10 mg/kg, i.p were studied in cotton pellet-induced granuloma model in wistar rats. Cotton pellets weighing 30±1 mg were autoclaved and soaked in 0.2 ml of distilled water containing penicillin (0.1 mg) and streptomycin (0.13 mg) and implanted subcutaneously bilaterally in axilla on ventral aspect of each rat. After 7 days rat was sacrificed and cotton pellets removed by dissection. It was weighed and recorded as wet weight. They were dried in hot air oven at 60°C for 24 hrs and weighed again. This was recorded as dry weight.

**Results:**

All three doses of rosiglitazone reduced the formation of granuloma tissue induced by cotton pellet method in a dose dependent manner. The percentage inhibition being 10 %,13% & 27% with doses 1,3 & 10mg/kg respectively. The reduction in granuloma formation with the dose of 10mg/kg was comparable with diclofenac sodium (positive control).

**Conclusion:**

In conclusion rosiglitazone has shown significant reduction in chronic inflammation in rats. These findings suggest that PPAR gamma agonists might be useful as therapeutic agents in the therapy of conditions associated with chronic inflammation.

**615/ AP04 G Achari Prize**

**A study of the rationality, pattern and economic implications of utilization of antimicrobial agents in the medical wards of a multispeciality tertiary level teaching hospital in Kolkata**

Ghosh M, Halder S, Bhattacharya K, Mondal S

**R. G. Kar Medical College, Kolkata, India.**

**Introduction:**

The use of ATC-DDD methodology has enabled drug utilization research to assess the pattern of drug utilization in a more objective way. As a developing country with a large and growing population, India is one of the significant consumers of antimicrobial agents (AMA). This study aims to assess the rationality, pattern and economic implications of AMA use in a state-sponsored setup.

**Methods:**

A retrospective study was conducted using the Inpatient case records of 100 consecutive patients and WHO ATC-DDD methodology to collect data regarding use of AMA in January 2007. Relevant investigation reports were also collected.

**Results:**

At least one AMA was prescribed in 83% patients, Ceftriaxone (J01DD04) being the commonest. 62% patients received parenteral AMA and the average parenteralto- oral switch time was 4.41 days. Empirical AMA therapy was initiated in 83%, streamlining and review of AMA regimen was performed in 15.6% cases but investigations supporting continued AMA use was available for 34.9% of the patients. The Mean±SD cost of AMA use to the patients was Rs 724.80±711.72, free-ofcost treatment was allowed to 35% of the patients admitted to the medical inpatient wards while hospital-supplied free-of cost AMAs were utilized in 55% of the study sample.

**Conclusion:**

Rational and cost-effective AMA utilization was observed for a small fraction of the patients studied, but in view of possible sampling errors, a larger study is proposed and pending approval of the Institutional Ethics Committee.

**616/ AP05 G Achari Prize**

**To study the effect of ethanolic extract of fruits of *Fragaria vesca* in experimentally induced inflammatory bowel disease**

Kanodia L, Das S

**Assam medical college, Dibrugarh, India.**

**Introduction:**

Ulcerative colitis and Crohn’s disease are chronic recurrent inflammatory bowel disease (IBD) of unknown origin. Oxidative stress is believed to be a key factor in the pathogenesis of the mucosal damage in IBD. Fragaria vesca (Wild strawberry), belongs to the family rosaceae. It has got mild astringent, antioxidant, anti-inflammatory and anti-diarrhoeal properties.

**Methods:**

Ethanolic extract of Fragaria vesca (EFFV) fruits was prepared by percolation method and subjected to oral toxicity testing using OECD guidelines. Albino rats were pretreated orally for five days with 3% gum acacia in control, EFFV 500mg/kg in test and 5-aminosalisylic acid (5-ASA) 100mg/kg in standard groups. Colitis was induced by transrectal administration of 4% acetic acid on 5th day. All animals were sacrificed with ether overdose 48 hours after colitis induction and 10cm colon segment was resected from proximal end. Colon was weighted (for disease activity index) and scored macroscopically and microscopically after histological staining. Biochemical assessment included myeloperoxidase (MPO) and colonic catalase, glutathione & SOD (Super oxide dismutase) measurements. Statistical analysis was done using one way ANOVA followed by Dunnett’s multiple comparison tests.

**Results:**

EFFV showed significant (*P*<0.05) prevention of increase in colon weight and disease activity index along with decrease in macroscopic lesions score as compared to control group. Significant increase (*P*<0.05) was observed in the levels of MPO, glutathione, catalase and SOD. There was significant improvement (*P*<0.05) in the histological scoring also. Effect of EFFV was significantly less than 5-ASA (*P*<0.05).

**Conclusion:**

EFFV at 500mg/kg showed significant amelioration of experimentally induced IBD.

**617/ AP06 G Achari Prize**

**Neuroprotective potential of pioglitazone in global cerebral ischemic-reperfusion injury is attributed to reduction in oxidative stress and apoptosis**

Kaundal R K, Sharma S S

**National Institute of Pharmaceutical Education and Research (NIPER), Sec-67, SAS Nagar Mohali, India.**

**Objectives:**

To investigate the neuroprotective potential of a PPARγ agonist; pioglitazone, in global cerebral ischemic-reperfusion (IR) injury.

**Materials and Methods:**

Global cerebral ischemia was induced by bilateral carotid artery occlusion (BCAO) for 5 min followed by reperfusion for 96 h in male mongolian gerbils. Neurological damage was assessed by behavioral parameters and histology of brain sections. Oxidative stress was estimated by measuring malondialdehyde levels and apoptosis was assessed through TUNEL (Terminal deoxynucleotidyl transferase-mediated dUTP nick end labeling) assay. Pioglitazone was administered intraperitoneally (i.p.) during BCAO in single dose study and in multiple dose administration study, Pioglitazone (3 mg/ kg, i.p.) was administered 1h, 24h, 48h and 72h after BCAO.

**Results:**

Global cerebral IR injury results in selective CA1 hippocampal neurodegeneration along with neurological deficits and hyperlocomotion. It was also associated with oxidative stress and apoptosis as evident from increased malondialdehyde levels and TUNEL positive cells. Pioglitazone treatment during BCAO has significantly reduced this neurological damage as evident from reduction in neurological symptoms, hyperlocomotion and CA1 hippocampal neuronal damage along with the attenuation of oxidative stress and apoptotic DNA fragmentation. Pioglitazone post ischemic multiple treatment has significantly reduced the CA1 hippocampal neuronal damage and DNA fragmentation after cerebral IR injury in IR challenged gerbils.

**Conclusion:**

This study demonstrates the neuroprotective activity of pioglitazone in global cerebral IR injury and its neuroprotective effects may be attributed to reduction in oxidative stress and apoptosis.

**618/ AP07 G Achari Prize**

**Efficacy study of *Prunus amygdalus* (almond) nuts in management of scopolamine induced amnesia in rats**

Kulkarni K, Mulay G, Mengi S

**C. U. College of Pharmacy, Mumbai, India.**

**Objective:**

The cognitive disorders such as amnesia, attention deficit, and Alzheimer’s disease are emerging nightmares in the field of medicine as no exact cure exists for them, as existing nootropic agents (piractam, tacrine, metrifonate) have several limitations. The present study was undertaken to investigate the effect of Prunus amygdalus nuts on cognitive functions, total cholesterol levels and cholinesterase activity in scopolamine induced amnesia in rats.

**Materials and Methods:**

The paste of P. amygdalus nuts (PA) was administered orally at three doses (150, 300 and 600mg/kg) for 7 or 14 consecutive days to rats. Piracetam (200 mg/kg) was used as a standard nootropic agent. Learning and memory parameters were evaluated using elevated plus maze (EPM), passive avoidance and motor activity paradigms.

**Results:**

It was observed that PA at above mentioned doses after 7 day or 14 day administration significantly reversed scopolamine (0.4 mg/kg i.p.) induced amnesia as was evidence by decrease in transfer latency in elevated plus maze task and step down latency in passive avoidance task. PA reduced the brain cholinesterase activity in rats. PA also exhibited a remarkable cholesterol and triglyceride lowering property and slight increase in glucose levels in the present study. PA demonstrated and total proteins levels when compared to the control.

**Conclusion:**

Since diminished cholinergic transmission and increase in cholesterol levels appear to be responsible for development of amyloid plaques and dementia in Alzheimer patients, PA may prove to be a useful memory restorative agent. It would be worthwhile to explore the potential of this plant in the management of Alzheimer’s disease.

**619/ AP08 G Achari Prize**

**Bay 11-7082, a NF-KB inhibitor reverses functional and sensorimotor deficits in experimental diabetic neuropathy**

Kumar A, Sharma S S

**National Institute of Pharmaceutical Education and Research (NIPER), Mohali, India.**

**Objective:**

To Evaluate neuroprotective potential of BAY 11-7082, a NF-kB inhibitor on functional and sensorimotor deficits in diabetic neuropathy.

**Materials and Methods:**

Sprauge Dawley (250-280 g) rats were used after IAEC approval. Diabetes was induced using Streptozotocin (55 mg/kg, i.p.). After six weeks of diabetes rats were treated with BAY 11-7082 (3 mg/kg). At the end of 8th week various functional (motor nerve conduction velocity and nerve blood flow), sensorimotor (thermal and mechanical hyperalgesia and allodynia), biochechemical (MDA and GSH levels) and immunohistochemical (NF-kB p65 levels) changes were studied.

**Results:**

BAY 11-7082 treatment reversed various deficits associated with diabetic neuropathy. Two weeks treatment increased motor nerve conduction velocity and nerve blood flow as compared to vehicle treated diabetic rats. The treatment corrected the abnormal sensory perceptions which developed in the diabetic animals after 6 weeks of diabetic neuropathy. There was increase in tail flick latency in cold and hot immersion tests. Treated animals showed increased threshold in mechanical hyperalgesia and allodynia. One of the interesting aspects of BAY 11-7082 treatment was its effect on oxidative stress. It decreased the elevated MDA levels and restored depleted glutathione levels in sciatic nerve of treated animals. It also decreased NF-kB levels in nerve microsections of diabetic animals as evident from decrease in NF-kB(p65) positive cells in nerve microsections.

**Conclusion:**

The present study proves the involvement of NF-kB activation in diabetic neuropathy. We can speculate promising future of developing NF-kB inhibitors as therapeutic agents against diabetic neuropathy.

**620/AP09 G Achari Prize**

**Evaluation of rasna panchaka (indigenous drug) as oxidative stress down-regulator using *in vitro* and *ex vivo* system**

Manjula T^1^, T. Prasanna Krishna^1^, Ramesh Sharma^3^, Anil K Balapure^3^, S.Venkateswar^2^, B. Dinesh Kumar^3^

**^1^Tissue Culture Laboratory, NLAC,Central Drug Research Institute, Lucknow; ^2^Faculty of Technology, Osmania University, Hyderabad; ^3^Food and Drug Toxicology Research Centre, National Institute of Nutrition, Jamai Osmani P.O, Hyderabad, India.**

**Introduction:**

Number of plants rich in phytochemicals used traditionally is important source of potentially therapeutic agents and is becoming favorite medicaments among the masses.

**Objective:**

To validate the therapeutic benefits of traditional preparations using in vitro and ex vivo studies for antioxidant activity.

**Methods:**

Antioxidant properties of water, methanol and water plus methanol extracts of 5 Indian medicinal plants, traditionally used in the treatment of arthritis were evaluated by three different in vitro systems. LA assay (linoleic acid assay) and mitochondrial peroxidation assay for preventive effect and DPPH assay (1,1 diphenyl 2- hydrazyl picryl assay) for scavenging property. The evaluated antioxidant activity (both preventive and scavenging activity) was recorded in the samples and compared with the standard Vitamin C. The plant extracts having potential in vitro antioxidant activity were subjected Explant culture is one such method, which is sensitive, reliable, reproducible and is capable of mimicking the in-situ conditions used for the screening of antioxidant activity. Mouse liver was excised aseptically, cut into 1-2mm3 (1-2 mg each) cubes and cultured in serum free medium 199 (Sigma) at pH 7.4. In each petri plate, a fixed number (12-15) of liver explants were maintained in 5ml of medium 199 in a humidified CO2 incubator at 370C. Extracts varying from 1-10*μ*g/ml were added to the culture plates. The antioxidant property was assessed by determining the primary oxidative defense enzymes viz. SOD, Catalase, GSH and MDA to determine the dose-response optima from 2-48 h period in mouse liver explants.

**Results:**

It was found from the In vitro tests study that water and water plus methanol extract’s of Rasna panchaka and water, methanol extracts of Ricinus communis have 5, 15, 3 and 4 fold higher activity compared to Ascorbic acid at the same level of concentration based on IC50 values. The results indicated that the cellular architecture of the cultured tissue was well conserved in the first 6h with gradual display of specific changes in the next 24h. There was a significant increase in MDA levels in experimental groups indicating the oxidative stress induction in explants. The explants showed significant protection against oxidative stress when incubated with extracts at the dose of 2*μ*g/ml. Lipid peroxidation was measured as malonaldialdehyde (MDA) reduced by 50%. This effect was accompanied by the increase in the first defense enzymes SOD (50%) and catalase (18%) with no change in reduced glutathione levels.

**Conclusion:**

The biologically significant role of these formulations as evident from the results of the present investigation not only validate the potential strength of the traditional medicine but also re-enforces the plausibility of their use as potent antioxidants.

**621/ AP10 G Achari Prize**

**Effect of rimonabant in overweight patients: A comparative study**

Samal R, Mohanty M, Mishra B R

**Department of Pharmacology; S.C.B. Medical College; Cuttack; Orissa; India.**

Rimonabant is a selective cannabinoid-1 receptor (CB1) blocker, that has shown to reduce body weight, thereby improves cardiovascular outcome. We randomly assigned 23 patients to take rimonabant 20mg daily whose body mass index (expressed as weight in kg divided by square of the height in meters) was between 30 to 35, who were under conventional treatment for other coronary risk factors. An age, sex matched control group of 11 patients were taken with similar BMI who were not assigned to take Rimonabant but were on conventional therapy for other risk factors. As compared to the control group Rimonabant at a dose of 20mg was associated with a significant (*P*<0.05) mean weight loss (-6.5±0.54kg) and reduction in waist circumference (-4.8±0.61 cm). Rimonabant also resulted in improvement of lipid profile. HDL cholesterol increased in treated group (9.3±2.4%) and triglyceride decreased (13.2±4.1%). However, changes in lipid profile were not shown to be independent of weight reduction. It was concluded that selective CB1 receptor blockade with Rimonabant significantly decreases body weight and waist circumference, however the improvement in lipid profile was not independent of weight reduction (r=0.08 n.s).

**622/ AP11 G Achari Prize**

**Evidence for involvement of corticotropin-releasing factor (CRF) 2 receptors in acute, chronic and withdrawal responses of nicotine on feeding behavior in rats **

Upadhya MA, Nakhate KT, Kamdi SP, Dandekar MP, Kokare DM, Subhedar NK

**Department of Pharmaceutical sciences, R. T. M. Nagpur University, Amravati Road, Campus, Nagpur (M. S.), India.**

**Objective:**

To investigate the role of corticotropin-releasing factor (CRF) type 2 receptors, within the framework of paraventricular nucleus of hypothalamus (PVN), in nicotine-induced anorexia.

**Materials and Methods:**

Male, Sprague Dawley rats were bilaterally cannulated targeting the PVN. CRF2 receptors agonist, CRF or antagonist α-hCRF_(9-41)_ and astressin_2_ β were administered alone or in combination with nicotine, 15 min prior to the commencement of dark cycle. Cumulative food intake was measured at 2 and 4 h post-injection time-points. Moreover, chronic and withdrawal effects of nicotine (4 mg/kg/day), alone or in combination with CRF (10 pM/rat, intra-PVN), were investigated with reference to daily food intake.

**Results:**

Although acute nicotine (2-4 mg/kg, intraperitoneal/ip), or CRF (10-100 pM/rat, intra-PVN), treatment dose-dependently reduced the food intake, α-hCRF_(9-41)_ (1 nM/rat, intra-PVN) and astressin_2_ β (1 nM/rat, intra-PVN) were ineffective. While pre-treatment of CRF (10 pM/rat, intra-PVN) potentiated, α-hCRF_(9-41)_ and astressin_2_ β antagonized the anorectic effect of nicotine at both the time-points. While nicotine treatment (4 mg/ kg/day, ip) significantly decreased daily food intake from day 1-6, tolerance was observed from day 7 onwards. However, concomitant treatment of CRF (10 pM/rat, intra-PVN) and nicotine showed persistent anorexia, suggesting attenuation of tolerance. Moreover, acute injection of CRF (10 pM/rat, intra-PVN) prevented the hyperphagic effect observed following day 3 of nicotine abstinence.

**Conclusion:**

We suggest that the transient anorexia, progressive tolerance and withdrawal hyperphagia following nicotine treatment, at least in part, may be mediated by CRF, particularly via CRF2 receptors in the PVN.

**623/ GP 01 Gufic Prize**

**Effect of saponin rich fraction of *Bryophyllum pinnatum* lam. on ethylene glycol induced urolithiasis in rats**

Bhalodia P, Gandhi T R, Patel K V

**Anand Pharmacy College, Anand, India.**

**Objective:**

To evaluate the effect of saponin rich fraction of Bryophyllum pinnatum Lam. on ethylene glycol induced urolithiasis in rats.

**Materials and Methods:**

Ethylene glycol model (0.75 % in drinking water, for 28 days) was used for renal stone induction. In preventive ethylene glycol model, saponin rich fraction of Bryophyllum pinnatum Lam. (SBp) (20 mg/kg, p.o.) was given for 28 days. Cystone (750 mg/kg, p.o.) was used as standard drug. After completion of experiment urine (24 hours) and blood samples were collected. Kidney homogenate was prepared. Kidney was also used for histological study. Various physical parameters and crystalluria were measured. Urine and serum samples were analyzed for various stone forming promoters, inhibitors, kidney function and degree of oxidative stress.

**Results:**

In model control group there was significant increase in various promoters like calcium, oxalate, inorganic phosphate and uric acid in various biological samples. While levels of inhibitors like citrate and magnesium in urine were significantly lowered. SBp and cystone significantly prevented alteration in various stone forming promoters and inhibitors. Treatment of test and standard drugs also significantly prevented alteration in physical parameters, kidney function and oxidative stress.

**Conclusion:**

Treatment with SBp showed significant prevention in renal stone formation in rats.

**624/ GP 02 Gufic Prize**

**Effect of dykure, a polyherbal formulation on streptozotocin-nicotinamide induced type-II diabetes mellitus**

Koneri R^1^, Parikh H^2^, Balaraman R^2^, Pandey A S^3^

**^1^Visveswarapura Institute of Pharmaceutical Sciences BSK 2nd Stage,Bangalore; ^2^Pharmacy Department, The M.S.University of Baroda, Vadodara, India; ^3^Herbal Body Cure Pvt. Ltd., C-278, Defense Colony, New Delhi, India.**

**Introduction:**

This study was undertaken to evaluate the effect of Dykure, a polyherbal formulation on diabetes and some associated metabolic abnormalities.

**Materials and Methods:**

Dykure was screened for hypoglycemic activity in normal and streptozotocinnicotinamide induced diabetic rats. Dykure was orally administered in two different doses of 300mg/kg and 1gm/kg. The various parameters studied included fasting serum glucose levels, serum lipid levels, liver glycogen content, and glycosylated hemoglobin HbA1c in diabetic and normal rats. Oral glucose tolerance was also studied.

**Results:**

Dykure produced significant hypoglycemic activity at both doses in diabetic rats. The body weight of diabetic rats significantly increased with all tested doses of Dykure. Treatment with Dykure at two dose levels showed a significant increase in the liver glycogen and a significant decrease in fasting blood glucose and glycosylated hemoglobin levels. The total cholesterol and serum triglycerides levels were also significantly reduced and the HDL cholesterol levels were significantly increased upon treatment with Dykure thus proving the potent antidiabetic and hypolipidemic property of this formulation.

**Conclusion:**

Dykure may be useful in the treatment of diabetes mellitus and associated metabolic abnormalities.

**625/ GP 03 Gufic Prize**

**Hypolipidemic effect of fresh *Triticum aestivum* grass juice in hypercholesterolemic rats**

Kothari S., Jain A.K., Mehta S.C., Tonpay S.D

**Gajara Raja Medical College, Gwalior, India.**

**Objective:**

Present study was aimed to elucidate hypolipidemic effect of fresh Triticum aestivum (common Wheat) grass juice (GJ) in experimentally induced hypercholesterolemic rats and to investigate presence of bioactive compounds in fresh GJ.

**Materials and Methods:**

Hypercholesterolemia was induced experimentally in rats by including 0.75gm% cholesterol and 1.5gm% bile salts in normal diet for 14 days. Hypercholesterolemic rats were administered fresh Triticum aestivum GJ at the dose of 5ml/kg and 10 ml/kg and the standard drug atorvastatin 0.02% w/v in 2% gum acacia suspension at the dose of 1mg/kg for 21 days by gavage. Blood samples were collected after 24 hours of last administration and used for estimation of lipid profile. Fecal cholesterol levels were estimated using standard methods. Fresh GJ was subjected to phytochemical analysis to find bioactive compounds.

**Results:**

Fresh GJ administration at 5ml/ kg and 10ml/kg resulted in significant decline in total cholesterol (TC), triglycerides (TG), low density lipoprotein-cholesterol (LDL-C) and very low density lipoprotein-cholesterol (VLDL-C) levels in hypercholesterolemic rats. Further in comparison to atorvastatin, GJ administration at the dose of 10ml/kg resulted in comparable decrease of TC and LDL-C levels and dose dependent significant (*P*<0.05) decrease of TG and VLDL-C levels. Fecal cholesterol excretion was significantly (*P*<0.05) enhanced by Triticum aestivum GJ administration. Phytochemical study of GJ revealed the presence of compounds that are known to decrease lipid level.

**Conclusion:**

The results of present study revealed hypolipidemic effect of Triticum aestivum GJ in hypercholesterolemic rats by increasing fecal cholesterol excretion. Additionally, fresh GJ contain hypolipidemic compounds that could have potentially beneficial effect in atherosclerosis associated with hyperlipidemia.

**626/ GP 04 Gufic Prize**

**Suppression of NF-KB signaling pathway by tocotrienol can prevent diabetes associated cognitive deficits**

Kuhad A, Chopra K

**Panjab University, Chandigarh, India.**

**Objective:**

Diabetes mellitus produces numerous neurophysiological and structural changes in the brain and it is associated with moderate cognitive deficits. The etiology of diabetes associated cognitive decline is multifactorial and involves insulin receptor down regulation, neuronal apoptosis and glutamatergic neurotransmission. The study was designed to evaluate the impact of tocotrienol mixture on cognitive function and neuroinflammatory cascade in streptozotocin-induced diabetes.

**Research Design & Method:**

Streptozotocin-induced diabetic rats were treated with tocotrienol (25, 50 & 100 mg/kg, orally) or with vehicle for 10 weeks. Morris water maze was used for behavioral assessment of memory. Cytoplasmic and nuclear fractions of cerebral cortex and hippocampus were prepared for the quantification of acetylcholinestrease activity, oxidative-nitrosative stress (lipid peroxidation, superoxide dismutase, catalase, non protein thiols, total nitric oxide), TNF-α, IL-1B, p56 subunit of NFκB and caspase-3.

**Results:**

After 10 weeks of steptozotocin injection, the rats produced significant increase in transfer latency which was coupled with enhanced acetylcholinestrease activity, increased oxidative-nitrosative stress, TNF-α, IL-1B, caspase-3 activity in cytoplasmic lysate and active p65 subunit of NFκB in nuclear lysate of cerebral cortex and hippocampus regions of diabetic rat brain. Interestingly, co-administration of tocotrienol significantly and dose-dependently prevented behavioral, biochemical and molecular changes associated with diabetes. Moreover, diabetic rats treated with insulin-tocotrienol combination produced more pronounced effect on molecular parameters as compared to their per se groups.

**Conclusions:**

Collectively, the data reveal that activation of NFκB signaling pathway is associated with diabetes induced cognitive impairment and point towards the therapeutic potential of tocotrienol in diabetic encephalopathy.

**627/GP05 Gufic Prize**

**Antioxidant effect of allylpyrocatechol in patients with rheumatoid arthritis**

Mitra A, Manna A, Kundu S, Saha P, Ganguly S, Sarkar A, Sen R, Sarkar D, Hariharan C, Seal K, Kumar K, Ghosh P, Dhar S, Ghosh A, Chatterjee M

**Institute of Post Graduate Medical Education & Research (IPGME&R), kolkata, India.**

**Objective:**

Rheumatoid arthritis (RA) is a chronic inflammatory disease where it is anticipated that antioxidants could play a supportive role in its management. Accordingly, the present study was designed to identify the antioxidant potential of Allylpyrocatechol (APC) obtained from an ethanolic extract of Piper betle linn. (Pann) in RA patients.

**Materials and Methods:**

Study population included naive cases of RA along with age, sex matched healthy individuals. The generation of reactive oxygen species (ROS) was measured using 2‘7’ dichlorofluorescin diacetate (H2DCFDA) in both erythrocytes and neutrophils by flow cytometry; results were expressed as geometric mean fluorescence channel (GMFC). The scavenging activity of APC was measured flowcytometrically in erythrocytes following incubation with H2O2 (0.5 mM). Its effect on phorbol 12-myristate 13-acetate (PMA,10 nM) induced oxidative burst in neutrophils was similarly measured.

**Results:**

The baseline ROS in patients with RA was 47% higher than healthy individuals. Addition of APC (0.25 and 0.5 *μ*g/ml) decreased GMFC in RA patients by 58% and 66% respectively whereas in healthy individuals, the decrease was lower being 28% and 44% respectively. In RA patients, H2O2 increased GMFC in erythrocytes by 45% as compared to healthy individuals which was effectively scavenged by APC. APC (2.5 and 5.0 *μ*g/ml) also decreased PMA-induced oxidative burst in neutrophils by 66% and 87% respectively

**Conclusion:**

Allylpyrocatechol (APC) showed promising antioxidant activity in erythrocytes and neutrophils of patients with Rheumatoid arthritis thus meriting further pharmacological investigations.

**628/ GP 06 Gufic Prize**

**Effect of ethanolic extract of *Mimosa pudica* leaf on wound healing in streptozotocin induced diabetic rats**

S Mohapatra, B Rath, A Panigrahi, S K Ranjit, C S Moharana, Y Roja Ramani, S Pradhan

**M.K.C.G. Medical College, Berhampur. India.**

**Aim and Objective:**

To study the healing potential of ethanolic extract of Mimosa pudica leaf (MLE) on incision and excision wounds in streptozotocin induced diabetic albino rats.

**Methods:**

For each wound model, twenty-five wistar albino rats (150-200 g) of either sex, were divided in to five groups (n=5). Diabetes was induced by streptozotocin (55 mg/kg i.p). Animals showing hyperglycemia ≥ 250 mg/dl were inflicted with incision/excision wounds and randomly divided. Gr I served as nondiabetic control, Group II received simple ointment (control). Gr III, IV and V rats were treated with framycetin sulphate -1%, MLE -2.5% & 5% ointment (topically) respectively. In the case of incision wound model, the treatment was given once a day for 10 days, whereas, the same treatment schedule was continued till complete healing of excision wounds. The healing parameters were assessed by measuring the skin breaking strength, histopathological evaluation for incision & rate of wound contraction, period of epithelialisation for excision wounds.

**Results:**

In incision wound model only MLE - 5% showed significant increase in skin breaking strength with supporting histopathological parameters, whereas in excision wound model, MLE with both the test doses (2.5% & 5%) showed significant responses in terms of decreased epithelialisation period & increase percentage of wound contraction when compared with the diabetic control group.

**Conclusion:**

The above findings suggest that ethanolic extract of Mimosa pudica leaf promoted wound healing in both the wound models in diabetic rats.

**629/ GP 07 Gufic Prize**

**Antianaphylactic activity of alcoholic extract of *Eclipta alba***

Patel MB^1^, Patel JA^2^

**^1^Shri Sarvajanik Pharmacy College, Mehsana, Gujarat, India;**

**^2^Institute of Pharmacy, Nirma University of Science & Tech. Ahmedabad, Gujarat, India.**

**Objective:**

To evaluate the Antianaphylactic Activity of Alcoholic Extract of Eclipta Alba in various experimental models.

**Materials and Methods:**

The Antianaphylactic activity of alcoholic extract of Eclipta alba with two different doses 250 mg/kg and 500 mg/ kg was studied by using different animal models like effect on mast cell degranulation using rat mesentery, passive cutaneous anaphylaxis using rat, by measuring leakage of Evans blue dye in skin, passive paw anaphylaxis using rat by measuring the paw volume by plethysmometer, Bronchoalveolar lavage fluid study in guinea pig trachea, measurement of different blood cells and level of histamine in lung tissues was performed.

**Results:**

Treatment with alcoholic extract of Eclipta alba showed a dose dependent beneficial effect on degranulation of mast cells in rats when challenged with Compound 48/80. Further a dose dependent beneficial effect was also observed on leakage of Evans blue dye in skin challenged with antigen. Eclipta alba showed beneficial effect on paw anaphylaxis induced by antiserum and also on infiltration of various inflammatory cells as well as on histamine release from lungs.

**Conclusion:**

Antianaphylactic activity of alcoholic extract of Eclipta alba may be possibly due to its membrane stabilizing potential, inhibition of antigen induced histamine release and inhibition of release of various inflammatory mediators.

**630/ GP 08 Gufic Prize**

**Evaluation of anti-plasmodium effect of leaf extract of *Nyctanthus arboi-tristis in mice infected with Plasmodium berghei***

Pradhan S, Mohanty S S, Y R Ramani, Panigrahi A, Mahapatra S

**Gajara Raja Medical College, Gwalior, India.**

**Aim and Objective:**

Malaria a dreadful disease, caused by four different parasites (Plasmodium falciparum, Plasmodium vivax, Plasmodium malariae and Plasmodium ovale), causing 1–3 million deaths each year. However increasing resistance to most presently available antimalarial drugs is a major obstacle to control this disease, if not eradicate it. In this work, the pharmacological potential of Nyctanthes arbortristis leaf extract was evaluated for in vivo inhibition of rodent malaria parasite species P. berghei.

**Methods:**

A rodent malaria parasite, Plasmodium berghei, which was maintained at the National Institute Of Malaria Research (Delhi) laboratory, was inoculated into Swiss albino mice. The mice were infected with 1×107 parasites intraperitoneally. The antiplasmodium effect of leaf extracts Nyctanthes arbortristis (100, 200, 400mg/kg) were evaluated by two methods; (1) 4- day supression test, where Chloroquine (5mg/Kg) was taken as standard and, (2) Prophylaxis study where proguanil (1mg/Kg) was taken as standard. The control groups received the same amount of solvent (vehicle) used to suspend each dose of the herbal drug. All the test agents were given intra gastrically.

**Results:**

Extracts from the leaves of Nyctanthes arbortristis inhibited Plasomodium berghei parasitaemia in the Swiss albino mice by 83.26% suppression of parasitemia level in 4-day supression test, and 58.30% in prophylaxis study.

**Conclusion:**

The study could partly confirm the claim in traditional medicine that the plant has therapeutic values in human malaria. There is, thus, the need to initiate further in-depth investigation by using different experimental models, and clinical trials.

**631/GP 09 Gufic Prize**

**Hypoglycemic action of ethanolic extract of leaves of *Oxalis corniculata* Linn. on normal and alloxan-induced diabetic albino rats**

Sarma G, Das S

**Assam Medical College, Dibrugarh ,India.**

**Introduction:**

Oxalis corniculata L., belonging to the family Oxalidaceae, is a common weed found throughout India. The objective of the present study is to evaluate the hypoglycemic action of ethanolic extract of leaves of Oxalis corniculata L. on normal and alloxan induced diabetic albino rats.

**Methods:**

30 healthy albino rats (Rattus Norvegicus) weighing 100-200 gms were randomly divided into 4 groups of six animals each. Group A and Group B received 10ml/kg/day of normal saline ; Group C received ethanolic extract of leaves of O. corniculata 500mg/kg/day; Group D received Glibenclamide 0.5mg/kg/day; all were given orally for 2 weeks. Alloxan (150mg/kg body weight) single dose was administered intraperitoneally to Groups B,C and D to induce diabetes. Blood sugar was estimated every week for 2 consecutive weeks along with body weight monitoring. For evaluation of mechanism of action of test drug, glycogen estimation was done in liver,heart and skeletal muscle and effect on adrenaline induced hyperglycaemia was seen. For hypoglycemic action on normal rats, blood sugar was estimated at ‘0’min and ‘120’min.

**Results:**

Group B showed significant (*P*<0.001) increase in blood sugar as compared to Group A. In comparison to Group B, Groups C and D showed significant (*P*<0.001) decrease in blood sugar. For mechanism of action, it was found that the test drug produces significant increase in liver glycogen. The test drug also significantly reduced adrenaline induced hyperglycaemia. Significant (*P*<0.001) lowering of normal blood sugar was also found.

**Conclusion:**

Ethanolic extract of leaves of Oxalis corniculata has significant antidiabetic and hypoglycemic activity.

**632/GP 10 Gufic Prize**

**Evaluation of antiurolithiatic activity of *Citrus medica* Linn. in rats implanted with calcium oxalate seed**

Shah A, Patel S, Patel K, Gandhi T

**Anand Pharmacy College, Anand, India.**

**Objective:**

To evaluate antiurolithiatic activity of Citrus medica using rats.

**Materials and Methods:**

Male Wistar albino rats (250-300g) were selected and divided into five groups. Except group I (normal) and group-II (Sham operated), in group-III (model control), group-IV (standard) and group-V (test) urolithiasis was induced by calcium oxalate seed deposition in bladder surgically (3 mm diameter). Cystone (750 mg/kg, p.o.) and C. medica (250 mg/kg, p.o.) were administered to group-IV and group-V for 14 days after surgery respectively. After the completion of treatment, blood and 24 hr urine samples were collected. Various physical parameters (body weight, water intake, diuresis, pH), stone forming promoters (calcium, oxalate, inorganic phosphate, uric acid) and inhibitors (magnesium, citrate) were analyzed. X-rays were taken before and after the treatment. Urinary bladder was opened and percentage growth of stone matrix was calculated. Stones were subjected to Scanning Electron Microscopy (SEM) and stereomicroscopy.

**Results:**

X-ray analysis revealed significant crystal growth in model control group. Significant increase in % matrix growth, stone forming promoters and decrease in physical parameters and stone forming inhibitors was observed in model control group. These changes were significantly prevented with the treatment of test and standard. SEM and stereomicroscopy results showed decrease in the stone growth, change in shape and surface of stone in treated groups as compared to model control group.

**Conclusion:**

This antiurolithiatic activity of C. medica. may be because of its ability to increase inhibitors’ level, decrease promoters’ level and promoting change in stone shape and texture.

**633/GP 11 Gufic Prize**

**Evaluation of the effect of *Quercus infectoria* olivier (fagaceae) in experimentally induced inflammatory bowel disease in rats**

Solanki R, Gandhi TR, Patel KV

**Anand Pharmacy College, Anand, India.**

**Objective:**

To evaluate the effect of Quercus infectoria olivier (fagaceae) in experimentally induced inflammatory bowel disease in rats.

**Materials and Methods:**

Sprague Dawley rats (200-300 gm) of either sex were randomly allocated to 6 groups (n=6). Except Group I (normal) and II (vehicle control) colitis was induced on 11th day by N-ethylmaleimide (NEM, 3%, 0.1 ml, intrarectally) in animals of groups III (model control), IV (std), V (test-300 mg/kg p.o) and VI (test-450 mg/kg p.o). 5 Amino salicylic acid (100 mg/ kg p.o), Quercus infectoria (300 mg/kg)and Quercus infectoria (450 mg/kg) was administered to groups IV, V and IV respectively for 18 days orally. During the study, animals were observed for various physical parameters. On 18th day of study rats were sacrificed, colon was scored histologically and various antioxidant parameters were measured in isolated colon tissue.

**Result:**

NEM caused increase in physical parameters like food intake, water intake, % body weight, and decrease in colon weight & length and stool consistency. Various oxidative stress parameters like MDA, NO, MPO levels in colon tissue homogenate were increased & SOD level was decreased by NEM. Treatment with 5 Amino salicylic acid , Quercus infectoria significantly prevented the changes induced by NEM in physical and oxidative stress parameters There was also significant improvement in histological scoring like colon mucosal damage index(CMDI), disease activity index(DAI), microscopic scoring, macroscopic scoring & histopathology of treatment groups as observed in group III.

**Conclusion:**

The results of our study suggest that Quercus infectoria therapy has beneficial effects on the course of experimental colitis.

**634/GP 12 Gufic Prize**

**Efficacy of sea buckthorn (*Hippophae rhamnoides* L.) On acute and chronic diabetic and burn wounds in albino rats**

Nitin K Upadhyay^1^, Ratan Kumar^1^, M S Siddiqui^2^, Asheesh Gupta^1^

**^1^Defence Institute of Physiology & Allied Sciences, Timarpur, Delhi-110054. ^2^Jamia Hamdard, Hamdard Nagar, Delhi-110062, India.**

***Hippophae rhamnoides* L**. (family: Elaeagnaceae), commonly known as sea buckthorn, is a wild shrub growing at high altitude (1200-4500 meters) in adverse conditions. The aim of the present study was to evaluate the healing potential of seabuckthorn extract (DIP-42) on rats using three different wound models (i) full thickness excision (ii) diabetic and (iii) burn wound model. After dose-response studies, the DIP-42 formulation was applied topically for 7 days. Control animals received the vehicle alone in identical manner. Povidone ointment for full thickness excision and diabetic wounds, and silver sulfadiazine for burn wounds were used as standard care. Wound granulation tissues were excised on day 8 postwounding, and the hydroxyproline, hexosamine, and total protein content were determined. Wound surface area was also measured on the eight day before wound excision to determine the wound contraction. In-vitro CAM angiogenic assay, differential protein expression (SDS-PAGE, gelatin zymography, and western blotting) and antioxidants profile were determined to find out the possible mechanism of action. The Sea buckthorn formulation promoted the wound healing activity as indicated by the findings of various pro-healing parameters. These finding were also confirmed by the histopathological examinations. Mechanistic studies showed that formulation also promoted angiogenesis and found to have potent antioxidant and wound debridement potentials. The results suggest that Sea buckthorn formulation (DIP-42) possesses significant wound healing potential.

**635/GP 13 Gufic Prize**

**Protective effect of green tea and vitamin E on biochemical and histopathological alteration in isoproterenol- induced myocardial infarction in rats**

Upaganlawar A, Balaraman R

**Faculty of Technology and Engineering, The M.S. University of Baroda, Vadodara, Gujarat, India.**

This study was designed to investigate the combined effect of green tea and vitamin E on serum marker enzymes, biomarkers of oxidative stress, heart lipid profile, membrane bound phosphatase and histopathological alteration during isoproteranol induced cardiotoxicity in rats. Male Albino rats sswere intoxicated with isoproterenol (200mg/kg, s.c) for 2 days at an interval of 24 hrs. resulting in marked elevation in heart weight, lipid peroxidation, serum marker enzymes (Serum glutamate oxaloacetate transaminase, Serum glutamate pyruvate transaminase, Lactate dehydrogenase, creatine kinase-MB), Lipid profile (Total cholesterol, triglyceride, free fatty acids and phospholipids) and Ca+2 ATPase levels where as a significant reduction in body weight, activities of endogenous antioxidants (Reduced glutathione, glutathion peroxidase, glutathione -s- transferase, superoxide dismutase and catalase), Na+/ K+ ATPase and Mg+2 ATPase levels. Treatment with vitamin E (100mg/kg/day, p.o) and/or green tea (100mg/kg/day, p.o) for 30 days and then intoxicated with isoproterenol on 29th and 30th days significantly attenuated these changes when compared to the individual treatment groups. Histopathological observations were also in correlation with the biochemical parameters. Thus, vitamin E and green tea significantly counteracted the pronounced oxidative stress effect of ISO by the inhibition of lipid peroxidation, restoration of antioxidant status, myocardial marker enzymes levels and lipid profile. In conclusion, these findings indicate the protective effect of vitamin E and green tea combination during ISO-induced cardiotoxicity and associated oxidative stress in rats.

**636/OP 01 O. D. Gulati Prize**

**Effect of nicorandil on blood glucose level in normal rats**

Biswas A, Mondal S

**R. G. Kar Medical College Kolkata, India.**

**Introduction:**

Oral nicorandil is occasionally used in middle aged and elderly patients for cardiovascular disorders. Many such patients also suffer from diabetes mellitus. Simultaneous use of nicorandil and oral sulfonylureas may interfere with the euglycemia. It was therefore felt necessary to study the effect of nicorandil in normal as well as in rats made hyperglycemic by glucose load and adrenaline injection.

The work was started after receiving permission from IAEC. Male albino rats between 90 and 120gm were divided into 4 control and several treatment groups with an average of six animals in each group. The controls were given the vehicle whereas the test groups were administered the study drugs. The blood samples were collected from lateral tail vein at an interval of 0hr, 1 hr, 2hr & 4hr and the blood sugar level was estimated by glucose oxidase method. The result was statistically analyzed by student ‘t’ test and one way ANOVA with Tukey’s& Dunnet’s post hoctest.

**Results:**

Nicorandil significantly increased the blood sugar level, reduced glucose tolerance and potentiated hyperglycemia in normal fasting rats, rats under oral glucose load and subcutaneous injection of adrenaline respectively. Nicorandil also significantly inhibited the hypoglycemic effect of glipizide in normalfasting rats.

**Conclusion:**

Nicorandil, though essentially a cardiovascular drug can increase blood sugar level and reduce glucose tolerance in rats within the human therapeutic dose range. As nicorandil inhibits the glipizide induced hypoglycemia it is appears to be acting by inhibiting the release of insulin by activating potassium channels in beta cells of islets of Langerhans. Diabetic patients simultaneously suffering from cardiovascular disorders receiving nicorandil may require dose adjustments of oral hypoglycemic agents.

**637/OP 02 O. D. Gulati Prize**

**A study of the adverse drug reactions in the inpatients of medicine department of Guru Gobind Singh Hospital, Jamnagar, Gujarat**

Vora M B^1^, Trivedi H R^2^, Tripathi C B^1^

**^1^Government Medical College, Bhavnagar, India; ^2^Shri M. P Shah Medical College, Jamnagar, India.**

**Introduction:**

Adverse drug reaction is now among the top ten leading causes of morbidity and mortality all over the world. There is a need to report the ADRs and to reduce the injury to the patients. My aim was to identify the life threatening ADRs in patients and to know the incidence rate of pre-hospital drugs used patients lead to ADRs and ADRs in hospitalized patients of General Medicine Wards.

**Materials and Methods:**

A prospective study of ADRs was carried out in General Medicine Wards, G.G.Hospital, Jamnagar. All patients of either sex and age >12 years admitted in General Medicine ward and patients fell in inclusion criteria were included in study. Patients of T.B. & Chest ward, Isolation ward, Dialysis unit, directly admitted in I.C.C.U. were excluded from study. During study, I visited all the patients of General Medicine wards and reported all patient related, drug related information in Case Report Form finalized in my study. If adverse drug reaction occurred, all details about ADRs were noted in Performa of ADR reporting form finalized in study. For statistical analysis, Chi square test was applied.

**Results:**

In this study, total incidence rate of ADRs was 5.42%. Among them 3.25% ADRs lead to hospital admission, 2.17% ADRs in already hospitalized patients of Medicine wards. Of all ADRs, 29.79% were life threatening ADRs.

**Conclusion:**

ADRs in hospitalized patients of General Medicine wards were less then pre-hospital drugs used hospitalized patients. Life threatening ADRs were more in already Hospitalized patients of General Medicine ward.

**638/OP03 O. D. Gulati Prize**

**Modulation of cardioprotective effect of ischemic preconditioning in hyperlipidaemic rat heart**

Yadav H N, Singh M

**I.S.F. Institute of Pharmaceutical sciences and Drug Research Moga-142001, Punjab, India.**

**Introduction:**

Inhibition of glycogen synthase kinase-3 beta (GSK-3 beta) is responsible for the cardioprotective effect of ischemic preconditioning. Moreover inhibition of GSK-3 beta produces inhibition of mitochondrial permeability transition pore (MPTP). The transgenic mice that overexpress GSK-3 beta, has been noted to develop hyperlipidaemia. The cardioprotective effect of ischemic preconditioning is attenuated during hyperlipidaemia in rats. Thus the present study has been designed to investigate the role of GSK-3β and MPTP in attenuation of ischemic preconditioning induced cardioprotection in hyperlipidaemic rats.

**Materials and Methods:**

Experimental hyperlipidaemia was produced in rat by feeding high fat diet for six weeks. The isolated Langendorff’s heart preparation, after stabilization for 5 min, was subjected to four episodes of ischemic preconditioning, each episode comprised of 5 min ischemia followed by reperfusion for 5 min. Then the heart was subjected to global ischemia for 30 min followed by 120 min of reperfusion. Myocardial infarct size was measured using TTC staining. LDH and CK-MB estimated in coronary perfusate. Lithium chloride (20 mM/l.) was administered after stabilization for 5 min followed by perfusion for 5 min with Kreb’s solution and four such episodes were repeated, then the heart was subjected for 30 min ischemia followed by 120 min reperfusion. Moreover Atractyloside was administered either in last reperfusion phase (for 5 min) of ischemic preconditioning or Lithium chloride (Licl) preconditioning.

**Result:**

The cardioprotective effect of ischemic preconditioning is attenuated in hyperlipidaemic rat heart. Moreover Licl preconditioning demonstrated cardioprotective effect in hyperlipidaemic rat heart. However Atractyloside attenuated cardioprotective effect of ischemic preconditioning in normal rat heart and Licl preconditioning in hyperlipidaemic rat hearts.

**Conclusion:**

Thus it may be concluded that attenuation of cardioprotective effect of ischemic preconditioning in hyperlipidaemic rat heart may be due to severe upregulation of GSK-3 beta which was inhibited by Licl.

**639/UP 01 U. K. Seth Prize**

**Efficacy and safety of diacerein in early knee osteoarthritis: A randomized placebo controlled trial**

Brahmachari B, Chatterjee S, Ghosh A

**Institute of Post Graduate Medical Education and Research (IPGMER) and SSKM Hospital, Kolkata, India.**

**Introduction:**

To evaluate efficacy and safety of Diacerein, an interleukin -1beta inhibitor, in the treatment of early, symptomatic knee osteoarthritis (OA).

**Materials and Methods:**

64 patients of either sex, aged between 35-70 years, diagnosed with symptomatic, early tibio-femoral compartment OA as per American College of Rheumatology Criteria, were randomized to receive either Diacerein (50mg OD for first 10 days and then BD) or identical looking placebo capsules for 8 weeks, followed by treatment free follow up of 4 weeks in this single-blind parallel group Phase IV trial. Primary efficacy variable was change from baseline in pain score assessed by VAS (Visual Analogue Scale) and secondary efficacy variables were changes in WOMAC subscores for stiffness and physical function, consumption of rescue medication (paracetamol) and Clinical Global Impression (CGI) assessed by the physician on a Likert scale.

**Results:**

55 evaluable subjects were analyzed for efficacy and safety parameters (28 in Diacerein; 27 in Placebo arm). Baseline demographic and disease profile characteristics were comparable between groups. Analysis of efficacy variables showed a highly significant reduction (*P*<0.01) in the VAS pain score and the WOMAC subscores for physical function and stiffness in the diacerein group. Consumption of rescue medication was significantly higher in the placebo group compared to diacerein group. Safety analysis showed transient changes in bowel habit in Diacerein group.

**Conclusion:**

Diacerein is an effective and safe agent for treatment of symptomatic knee joint OA.

**640/UP 02 U. K. Seth Prize**

**Potential drug interactions with *Curcuma longa*: Neuropsychopharmacological studies on mice behavioral models**

Gohil K J^1^, Patel J A^2^

**^1^Maliba Pharmacy College, Gopal Vidhyanagar, Surat, India;**

**^2^Institute of Pharmacy, Nirma University of Science & Technology, Ahmedabad, Gujarat, India.**

**Introduction:**

Recently, the interactions of herbal medicine with synthetic drugs came in to focus of particular interest. Herbal medicine may cause significant toxicity with additive, synergistic or antagonistic effect when taken in combination with allopathic drugs.

**Objective:**

To investigate the potential interaction between curcuma longa (CLE) and reboxetine (RBX) on 2-week, chronic, oral, once daily, administration in mice.

**Materials and Methods:**

Male Swiss, albino, mice were divided in to eight groups of six each. Control group-I was administered distilled water. Group II received drug RBX 20mg/kg/day. Group III, IV and V received standardized water soluble CLE containing 0.5% of curcumin in doses of 140, 280 and 560 mg/kg/day respectively, while Groups VI and VII and VIII received combinations of RBX along with CLE in three different doses. Spontaneous motor activity (SMA), open field behavior, motor coordination, forced swimming test (FST) tail suspension test (TST) and chronic fatigue tests were evaluated. Biochemical parameters were checked in blood and brain samples.

**Results:**

Test groups VI, VII and VIII showed significant reduction in SMA, open field behavior,‘fall off’ time and immobility period in FST on 7th and 15th day and in 7-day chronic fatigue tests. No major changes were observed in immobility period in TST. Nor adrenaline turnover was significantly increased in combination treated groups compared to control and standard groups.

**Conclusion:**

There is a possibility of interaction between curcuma longa and RBX. Precautions are advised while using this combination. Potential interactions of curcuma longa with newer antidepressants are matter of further study.

**641/UP 03 U. K. Seth Prize**

**Effect of cilostazol on platelet aggregation in patients with unstable angina and non-ST elevation acute myocardial infarction**

Pattanaik Smita, Malhotra S, Sharma YP, Ahuluwalia J, Pandhi P

**Dept. of Pharmacology, PGIMER, Chandigarh, India.**

**Background:**

Antiplatelet drugs are the cornerstones of therapy in non-ST elevation acute coronary syndrome (NSTEACS) and include aspirin, clopidogrel and GPIIb/IIIa inhibitors. The most optimal antithrombotic regimen has not yet been defined and the risk of ischemic events remains high in these patients.

**Patients and Methods:**

Forty patients of NSTEACS presenting within 72 hours of onset of symptoms, were randomized to cilostazol or placebo in 1:1 ratio. Cilostazol 100mg b.i.d was administered within 12 hours of hospital admission in a dose of for 7 days in addition to the standard antiplatelet regimen of aspirin and clopidogrel. The primary end points were changes in the percentage platelet aggregation and serum plasminogen activator inhibitor (PAI-1). The secondary endpoint was a composite of recurrent ischemia, myocardial infarction, need for intervention and death at 30 days. Adverse drug events were recorded at 7 and 30 days.

**Results:**

Patients in the triple therapy group showed significant decrease in the ADP [27 (28.5) vs 5.3 (8.5); *P*=0.003] and epinephrine [40.7(21.8) vs 24.3(16.4); *P*=0.03] induced percentage platelet aggregation after 7 days of treatment compared to the dual therapy group. There was no change in levels of serum PAI-1 [50.30 (10.17)ng/ml vs 53.47 (14.08)ng/ml; *P*=0.42]. The composite end point occurred in 7 patients in the cilostazol group compared to 3 in the placebo group at the end of 30 days of follow-up [*P*=0.48]. No serious adverse events occurred during the study.

**Conclusion:**

Cilostazol has additive platelet aggregation inhibition action in patients with NSTEACS when added to aspirin plus clopidogrel.

**642/UP 04 U. K. Seth Prize**

**An *in vitro* study of effect of statins on normal lens and glucose induced cataract**

Y R Ramani, Panda A, Panigrahi A, Mohapatra S, Pradhan S

**MKCG Medical College, Berhampur, India.**

**Objectives:**

Cataract, a condition in which the lens becomes opacified, is one of the major complications of diabetes. Oxidative damage by the free radicals has been implicated in the pathology of catractogenesis. Diabetes with dyslipidemia is a common finding where statins have clearly established their role as 1st line drugs. The present study delineated the status of statins in normal lens and diabetic cataracts in an in vitro model of experimental cataract.

**Methodology and Techniques:**

Goat lenses obtained from local slaughterhouse were incubated in artificial aqueous humour containing 5.5 mM of glucose or 55 mM of glucose (catarctogenesis) with statins in different concentrations at room temperature for 72 hrs. Opacification of lens was assessed by photographic evaluation after 72 hrs of incubation. Biochemical parameters like cholesterol and MDA levels of lens homogenate were estimated. All the data relating to biochemical parameters were compared using one-way ANOVA with post-hoc Dunnet’s test.

**Results:**

Glucose induced opacification of lens started 12 hrs post incubation and was complete in 72hrs. Cataractous lenses showed higher cholesterol and MDA levels (*P*<0.01). Lenses treated with Atorvastatin or Simvastatin in concentrations of 15 and 60ng/ml showed higher protein (total and water soluble) content and prevented formation and progress of cataract by glucose as evidenced by biochemical parameters.

**Conclusion:**

The anticataract activity of Statins may be because of the antioxidant activity as evidenced by lower MDA levels in treated lenses.

**643/UP 05 U. K. Seth Prize**

**Study of the clinical efficacy and safety profile of imatinib mesylate, a novel tyrosine kinase inhibitor in the management of chronic myeloid leukemia**

Swain TR^1^, Rout N^2^, Mohanty M^1^

**^1^S.C.B Medical College Hospital, Orissa, India; ^2^AH Regional Cancer Centre, Orissa, India.**

Seventy patients diagnosed as chronic myeloid leukemia (CML) attending haematology OPD of SCB Medical College& hospital, Cuttack, were included in the present study. The data regarding dose, brand, duration, ADR profile of Imatinib was included in the predesigned format. Efficacy of Imatinib was noted by determining clinical and haematological parameters as well as estimating bcrabl transcript level done every 6 month by real time quantitative PCR assay which monitors kinetics of residual bcr-abl transcript over time. Failure to achieve major molecular response by 18 months after starting imatinib was considered to be suboptimal response requiring, reevaluaion, and reassessment of therapy. The result was also correlated with haematological response which includes DC, TLC,Hb,Peripheral blood smear analysis which was done by an approved haematology laboratory. Data regarding adverse effect of imatinib was recorded in the dept. of Pharmacology which include Weight,BP, CVS examination,pigmentation & psychological wellbeing of the patient etc which was measured on a graded scale. 65 out of 70 patients improved significantly in haematological response with imatinib at the dose of 400 mg/ day. Patients receiving Glivac free of cost (imatinib of Novartis under GIPAP) being maximally well. More serious form of ADR like bone marrow failure syndrome, pancytopenia was found in patients who started the treatment late. Mild initial ADRs like oedema, pigmentation and G.I disturbance improved significantly as the disease clone got inhibited. Imatinib Mesylate is quite efficacious in the management of CML patients with acceptable side effect if started early in the disease course.

**644/UP 06 U. K. Seth Prize**

**Study of comparative efficacy between nebulised magnesium sulphate with salbutamol and salbutamol alone in patients with acute exacerbation of chronic obstructive pulmonary disease**

Sarkar S, Ray K, Deb J, Mondal S

**R G Kar Medical College, Kolkata, India.**

**Objective:**

To test the hypothesis that combined administration of nebulised salbutamol and magnesium sulphate provides additional benefit compared with salbutamol alone in adult patients with acute exacerbation of chronic obstructive pulmonary disease.

**Methods:**

A randomized, open, prospective, parallel study comprising of 60 patients attending to Chest Medicine OPD with acute exacerbation of COPD. Group A received nebulisation with a combination of salbutamol and magnesium sulphate and Group B received nebulisation with salbutamol alone. Both the groups received a single dose of nebulisation. Salbutamol and magnesium sulphate were administered in doses of 0.5mg and 500mg, respectively and the solutions were made isotonic to plasma osmolality. Pulse rate, blood pressure, and peak expiratory flow rate (PEFR) were measured at baseline and at 45 and 90 min. Serum magnesium levels and Oxygen saturation were measured at 0 and 90 min in both groups.

**Results:**

The baseline parameters were comparable in both the groups. Both groups showed significant rise in PEFR at all time intervals (group A *P*=0.0001and group B *P*=0.0001) and also there was significant difference between the groups in rise in PEFR at any time (*P*=0.0001). Oxygen saturation were also raised in both the groups at time interval (group A *P*=0.0001 and group B *P*=0.0001). This evidenced significant difference between the groups (*P*=0.0001). Serum magnesium level were significantly increased in both the groups (group A *P*=0.0001 and group B *P*<0.001) within the normal limits, however, there was no difference between the groups. No notable side effects were noted.

**Conclusion:**

Administration of 500mg of nebulised magnesium sulphate reduces bronchoconstriction when used as adjunct to salbutamol in patients with exacerbation of COPD.

